# Corrigendum: Alleviation of Synovial Inflammation of Juanbi-Tang on Collagen-Induced Arthritis and TNF-Tg Mice Model

**DOI:** 10.3389/fphar.2020.01127

**Published:** 2020-07-24

**Authors:** Tengteng Wang, Qingyun Jia, Tao Chen, Hao Yin, Xiaoting Tian, Xi Lin, Yang Liu, Yongjian Zhao, Yongjun Wang, Qi Shi, Chenggang Huang, Hao Xu, Qianqian Liang

**Affiliations:** ^1^ Longhua Hospital, Shanghai University of Traditional Chinese Medicine, Shanghai, China; ^2^ Institute of Spine, Shanghai University of Traditional Chinese Medicine, Shanghai, China; ^3^ Second Ward of Trauma Surgery Department, Linyi People’s Hospital, Linyi, China; ^4^ Key Laboratory of Theory and Therapy of Muscles and Bones, Ministry of Education, Shanghai University of Traditional Chinese Medicine, Shanghai, China; ^5^ Shanghai Institute of Materia Medica, Chinese Academy of Sciences, Shanghai, China; ^6^ Department of Pathology and Laboratory Medicine, University of Rochester, Rochester, NY, United States

**Keywords:** Juanbi-Tang, rheumatoid arthritis, collagen-induced arthritis, synoviocyte, tumor necrosis factor

The authors found that **Figure 2F** was incorrect. During the first revision, the authors replaced the bar graph with a dot plot according to a reviewer’s suggestion; however, the authors missed **Figure 2F** and placed **Figure 2D** where **Figure 2F** should be placed. The correct **Figure 2F** appears below.

The authors apologize for this error and state that this does not change the scientific conclusions of the article in any way. The original article has been updated.

**Figure 2:**
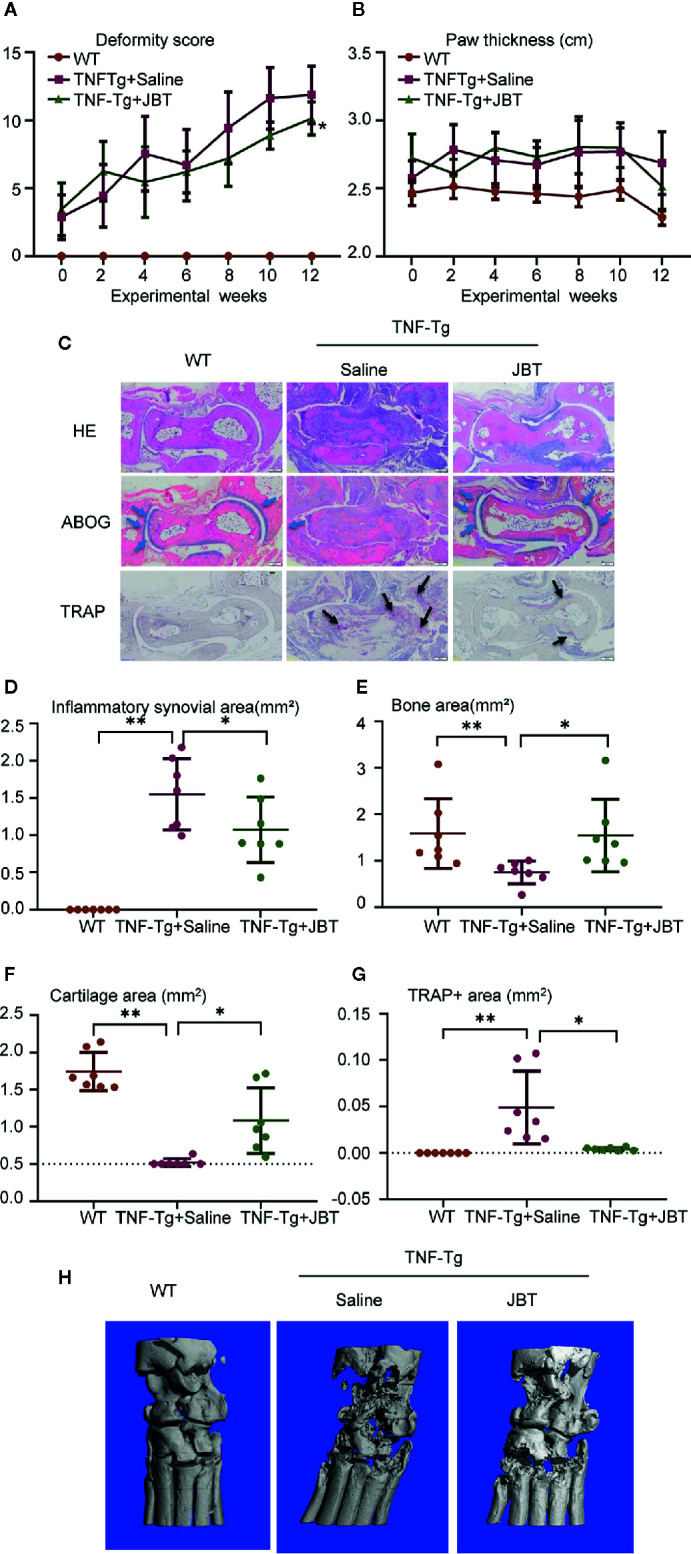
Juanbi-Tang (JBT) attenuated symptoms, inflammatory synovial volume and bone erosion at ankle joint of tumor necrosis factor transgenic (TNF-Tg) mice. The deformity score **(A)** and hind paw thickness **(B)** of TNF-Tg mice and WT littermates were scored and recorded every week in a blinded manner. **(C)** Hematoxylin–eosin staining, ABOG staining, and TRAP staining were used to analyze the ankle joints of WT and TNF-Tg mice, which were treated with saline or JBT for 12 weeks. Bar, 200 μm. **(D)** Histomorphometric assessment of inflammatory synovial volume area. **(E)** Histomorphometric assessment of bone area. **(F)** Histomorphometric assessment of cartilage area. **(G)** Histomorphometric assessment of TRAP+ osteoclast area. **(H)** Micro-CT of ankle joints. Results are shown as mean ± SEM, for 6–8 legs per group. Three sections per mice were analyzed. **p < 0.01 versus TNF-Tg + saline, *p < 0.05 versus TNF-Tg + saline.

